# Supplementary material for: Nuclear blebs are composed of variable chromatin states but consistently enrich transcription initiation relative to elongation
Source: bioRxiv. 2026 Mar 11:2026.03.10.710873. Preprint. [Version 1] doi: 10.64898/2026.03.10.710873 (PMC13060922; doi:10.64898/2026.03.10.710873)
Supplement: Supplement 1 [file media-1.pdf]

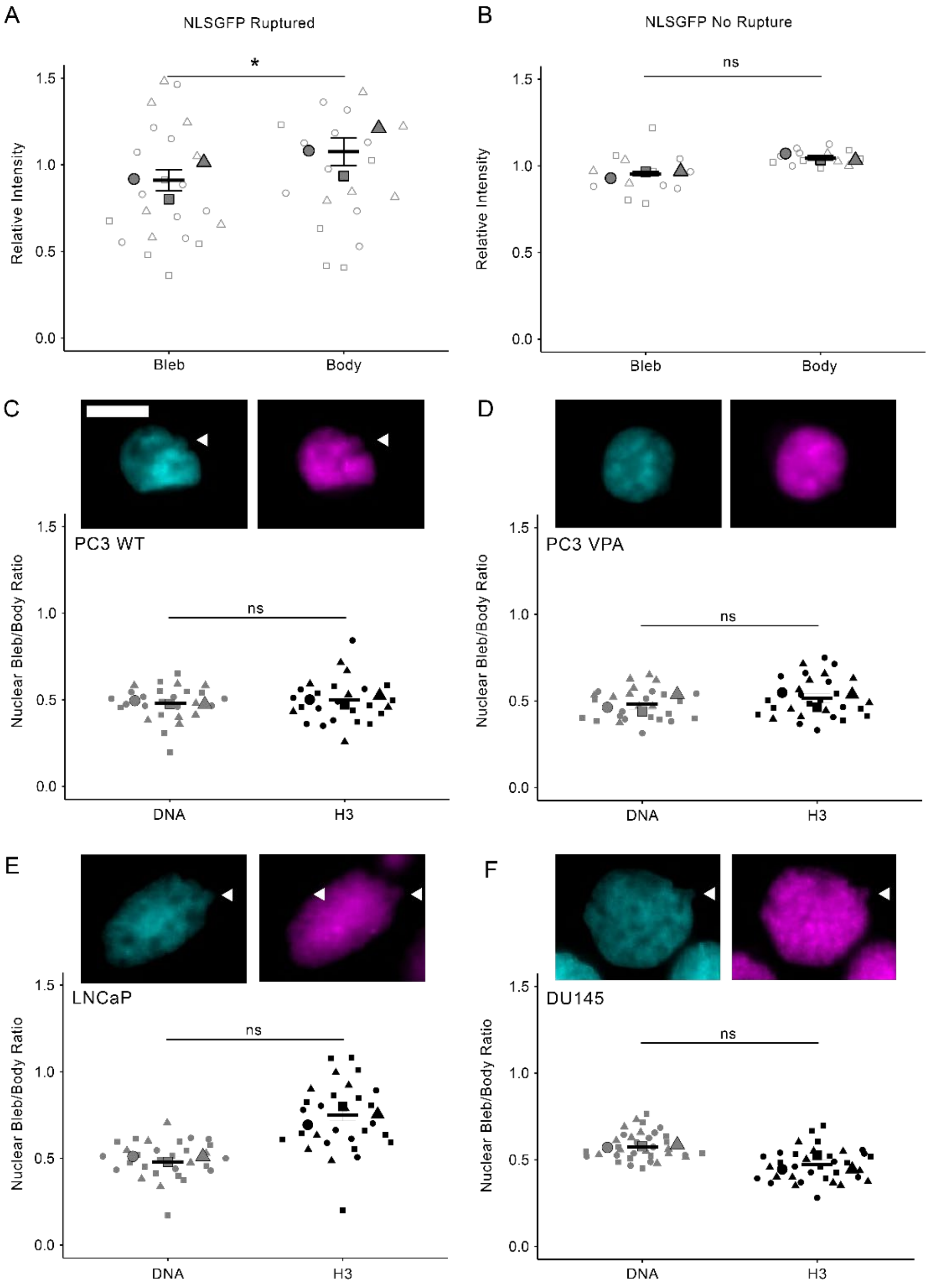

Supplemental Figure 1. Histone levels in the nuclear bleb relative to the nuclear body vary across human prostate cancer cell lines. Super plots of NLS-GFP relative intensity in the bleb and body for nuclei that (A) rupture, biological triplicates n = 22, or (B) three nuclei that do not rupture imaged every hour, n = 5, 5, 3. Data analyzed from Figure 1 A and B. (C-F) Example images and super plots of nuclear bleb to body ratio for Hoechst 33342 DNA stain (cyan) and H3 (magenta) for cell lines (C) PC3 n = 9, 10, 8, (D) PC3 VPA n = 10, 9, 10, (E) LNCaP n = 7, 10, 10, (F) DU145 n = 12, 12, 11. White arrows denote nuclear blebs. Statistical significance is denoted by *P<0.05, **P<0.01, ***P<0.001 or ns (not significant) via two-tailed paired Student's t-test. Scale bars: 10 μm.

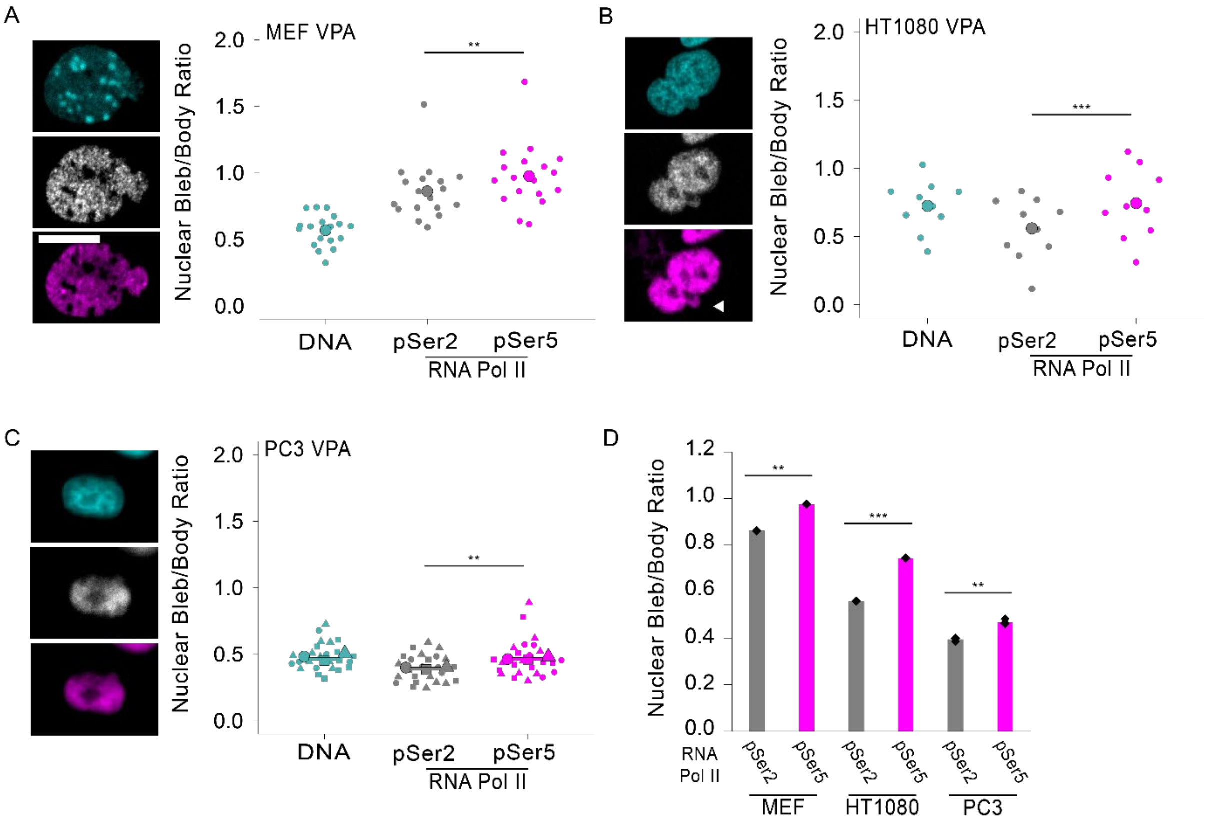

Supplemental Figure 2. Active RNA Pol II pSer5 initiation is enriched relative to pSer2 elongation in nuclear blebs of cells treated with VPA. Example images and super plots of nuclear bleb to body ratio for DNA (cyan) and RNA Pol II pSer 2 (gray, elongation) and pSer5 (magenta, initiation) for cell lines traeated with histone deacetylase inhibitor VPA 4 mM for 24 hours (A) MEF VPA n = 18, (B) HT1080 VPA n = 10, (C) PC3 VPA n = 6, 9, 10. (D) Graph summarizing all cell lines show a significant enrichment of RNA Pol II pSer5 relative to pSer2 in the nuclear bleb to body ratio. Statistical significance between pSer2 and pSer5 is denoted by *P<0.05, **P<0.01, ***P<0.001 or ns (not significant) via two-tailed paired Student's t-test. Scale bars: 10 μm.
